# Eye and Gaze Behavior During Human Disassembly Procedures: Effects of Task Conditions and Process Dynamics

**DOI:** 10.3390/jemr19040091

**Published:** 2026-08-18

**Authors:** Manuel Zaremski, Barbara Deml

**Affiliations:** Institute of Human and Industrial Engineering, Karlsruhe Institute of Technology, 76131 Karlsruhe, Germany; barbara.deml@kit.edu

**Keywords:** eye tracking, eye movements, gaze behavior, cognitive processes, task performance, skill level, human factors, disassembly, remanufacturing

## Abstract

Manual disassembly is a key process in remanufacturing and circular-economy systems, yet it is often characterized by uncertainty, varying product conditions and non-standardized action sequences. Eye tracking provides a process-oriented methodology for examining how such tasks are visually guided, with implications for human behavior analysis, operator support, and automation design. The present study investigated the potential of specific eye and gaze metrics to reflect differences in task condition and process dynamics during human disassembly. These were analyzed using a dimension-based mixed-effect modeling approach, accounting for task condition, handcraft skill levels, and task duration across repeated observations within participants, addressing four dimensions: visual attention allocation, cognitive workload, scanpath structure, and gaze organization. The results showed that increased task uncertainty was most associated with shorter fixation durations, higher fixation rates, larger pupil diameters, and higher revisit rates, indicating intensified visual sampling, increased cognitive workload, and repeated checking behavior. No robust differences were observed between skill levels. A longer task duration, as an indirect proxy of less routine-like execution, was primarily reflected in fixation dynamics and scanpath structure, rather than in entropy or workload measures. The findings demonstrate the sensitivity of eye and gaze metrics to task uncertainty and process dynamics in disassembly.

## 1. Introduction

The global transition towards sustainable industrial systems has become a central priority as the consequences of climate change, resource scarcity, and increasing waste accumulate [1]. Circular economy strategies aim to retain the long-term value of materials and extend product life cycles [2], with remanufacturing emerging as a key approach. This involves the reprocessing of used products to a condition comparable to new ones, at a substantially reduced energy and material cost. Despite its ecological and economic potential, remanufacturing remains challenging to operationalize, particularly regarding the non-destructive disassembly of used products [3,4]. Unlike standardized assembly processes, disassembly has to deal with variability in product configuration and condition, including degradation, damage, contamination, and unknown usage histories [5], complicating production planning and limiting the feasibility of fully automated solutions [6]. Disassembly therefore remains one of the most manual and knowledge-intensive phases of remanufacturing [7], where human expertise is crucial for process reliability and quality [8]. Understanding how human operators visually guide disassembly is therefore both practically and scientifically relevant. Manual disassembly is a visually demanding activity that requires continuous inspection, action planning, and adaptation to changing task demands [9]. Incomplete prior knowledge, variable product conditions, hidden constraints, and unexpected deviations from intended sequences mean that disassembly places unusually high perceptual and cognitive demands on the operator. Conventional performance measures, such as task duration, completion rate, and error frequency, are capable of capturing whether a task was completed successfully [10]. However, they offer limited insight into the underlying cognitive processes that guide action during execution [11,12]. While two operators may achieve similar outcomes, their visual strategies may differ significantly: one may inspect the workspace in a focused, anticipatory manner, while another may exhibit repeated checking, broad visual search, or unstable gaze behavior. Eye tracking provides a process-oriented perspective on these differences by offering insight into visual behavior [13].

Research across various domains, including visual search [14,15], industrial work [16,17], medicine [18], and behavioral studies [19,20], has demonstrated that eye and gaze metrics can serve as indicators of task difficulty, uncertainty, cognitive workload, expertise, and procedural organization. In manual task contexts, fixation- and saccade-based measures characterize attentional sampling and visual search [15], changes in pupil size serve as an indicator of cognitive workload [9], scanpath measures capture the spatial and sequential organization of gaze [21], and entropy-based indices quantify the regularity of gaze allocation and transitions [22]. By integrating these dimensions, a detailed understanding of the organization of visual behavior and insights into the cognitive and procedural demands during task execution can be achieved. Despite the relevance of these approaches, research into eye tracking in the context of disassembly remains limited in comparison with related domains such as assembly [17], visual inspection [8], or surgical action [18]. However, in the context of manual assembly and inspection, an area-of-interest-based eye-tracking approach was used to localize the concentration of cognitive workload and errors within a multi-step assembly task [23]. This showed that fixation duration and count differed systematically between the measuring and attaching subtasks and that gaze transitions between areas of interest deviated from the standard predicted path. In an applied usability context [24], a gaze-controlled instruction interface for manual assembly was benchmarked against a conventional paper manual in terms of both subjective workload measures and objective assembly time. This illustrates that gaze can be harnessed interactively and not only diagnostically during manual tasks. Furthermore, a review of eye-tracking applications in manufacturing and logistics settings [17] proposed a conceptual framework spanning process performance, human performance and workplace safety. While it was shown that eye tracking has been applied in manufacturing and logistics to study visual attention, workload, training, human error and human–system interaction, it was also emphasized that many applications remain concentrated on assembly, inspection, order picking and interface-related tasks rather than disassembly. However, there is a lack of research on how eye and gaze behavior varies across disassembly conditions and how this relates to variations in process dynamics. This discrepancy is of crucial significance, as disassembly is characterized by several factors, including uncertainty, procedural adaptation, re-inspection, and variable action fluency, which are hypothesized to exert the most substantial influence on visual behavior. Addressing this gap also carries broader industrial relevance: the identification of visual signatures of uncertainty, the monitoring of behavior, and the execution of routine procedures can inform the design of adaptive assistance systems and human–machine interaction, and may support the transfer of human procedural knowledge to robotic disassembly systems.

The present study addresses this gap by examining eye and gaze behavior during manual disassembly across varying task conditions and process dynamics. The selected metrics were analyzed across four conceptual dimensions: visual attention allocation, cognitive workload, scanpath structure, and gaze organization and entropy. This approach uses global process-level analysis of eye and gaze metrics rather than fine-grained determination of behavioral actions based on additional information about hand movements or object-specific interactions. The study was guided by two main research questions. Firstly, we investigated the effect of different disassembly conditions on eye and gaze behavior during human disassembly (RQ1). Secondly, we examined how process dynamics during disassembly are reflected in eye and gaze behavior (RQ2). The second question was explored through two sub-questions: whether eye and gaze measures differ between self-assessed skill levels (RQ2a) and whether they are associated with routine-like forms of task execution (RQ2b), as operationalized indirectly through task duration, skill level, and repeated trial execution. The investigation was conducted as a controlled laboratory study of eye and gaze behavior during manual disassembly. The primary focus of the present study was on task-condition effects and process dynamics across repeated disassembly trials. A secondary and more exploratory objective was to estimate a feasible comparison between skill levels and an indirect proxy for routine-like forms of task execution. The evaluation of skill level was predicated on self-assessed handcraft experience as opposed to verified professional disassembly expertise. Based on prior research on visual search and workload in action-oriented tasks [15,20], more demanding disassembly conditions were expected to be associated with more intensive visual sampling, increased inspection behavior, broader or less stable scanpaths, and greater pupil-based workload responses. With regard to process dynamics, higher skill levels were expected to be associated with more stable and structured gaze behavior. Longer task duration and repeated disassembly trials, used as an indirect indicator of less routine-like execution, were expected to be associated with more active visual search, increased revisiting, and less stable scanpath organization.

## 2. Eye and Gaze Metrics as Indicators of Manual Action Behavior

Eye tracking provides access to visual behavior that can be observed during the execution of a given task [12], and is therefore well suited to the analysis of manual action. In the context of disassembly, eye and gaze behavior assumes particular significance, as effective performance necessitates the continuous coordination of perception and action [11]. Participants are required to inspect components, monitor the current state of the workspace, identify relevant manipulation points, verify task progress, and adapt to uncertain or changing conditions. Consequently, different eye and gaze metrics [25] can facilitate the investigation of how manual action is visually guided and cognitively regulated.

Eye fixations represent sustained gaze on a specific location and follow systematic patterns that align with ongoing actions [26]. Research findings indicate that during manual tasks, workers focus on the object they are manipulating and scan task-relevant elements when planning more complex sequences [27]. Short fixations facilitate rapid object localization, whereas longer fixations are indicative of detailed feature processing and monitoring of the unfolding action. Furthermore, an increased fixation duration may indicate a reduced need for exploration rather than an inherent difficulty [28]. Saccades are defined as fast, short-term eye movements that guide the eyes to a novel spatial region of interest. Research findings [29,30] show that longer saccade duration and latency are typically associated with motor and fatigue constraints, higher cognitive load or less efficient control, while shorter, high-velocity saccades tend to mark more efficient, successful performance. The modulation of pupil size is a function of the autonomic nervous system, and is associated with emotional and cognitive arousal [31]. Variation in size is a characteristic measure that is utilized in research on mental or cognitive processes [19]. Additionally, eye blinks, defined as a rapid closure and opening of the eyelid, show similarity to emotional and cognitive processing, particularly with regard to attentional engagement and mental workload [19]. In the context of action execution, the frequency of blinks and the size of the pupils have been shown to serve as reliable indicators of cognitive load during automotive manufacturing tasks [9]. Gaze entropy is defined as a quantitative metric for measuring variable or predictable eye movements. Recent studies [32] have demonstrated the efficacy of gaze entropy as a means to infer the influence of implicit knowledge, learning, and action control on visual exploration. The distinction between stationary gaze entropy and gaze transition entropy allows for the reflection of visual scanning efficiency in complex, predictive gaze control [33]. Higher entropy scores are indicative of uncertainty-driven exploratory behavior [34], while lower entropy scores are indicative of implicit skills that are more automated [35].

Thus, to investigate the objectives of this study, eye and gaze behavior is interpreted as an objective indicator of the perceptual and cognitive processes underlying manual action. To capture different aspects of disassembly-related visual behavior in a theory-driven way, the selected eye and gaze metrics were organized into four conceptual dimensions (see Table 1) while avoiding an undifferentiated treatment of individual metrics as isolated outcomes.

The first dimension, visual attention allocation, describes how visual attention is distributed during task execution [36]. It was operationalized using mean fixation duration, fixation rate, and mean saccade amplitude. Mean fixation duration reflects the temporal allocation of attention to currently inspected information and is commonly interpreted as an indicator of local processing demand or attentional engagement. Fixation rate captures how frequently the visual environment is sampled and can therefore be interpreted as an indicator of search intensity and attentional updating. Mean saccade amplitude reflects the spatial extent of gaze shifts and provides information about whether visual exploration is more local or more broadly distributed across the workspace. Selection of these measures resulted from their ability to capture both temporal and spatial characteristics of visual search during disassembly. However, mean saccade duration and saccade rate were not included in the final selected metric set due to their lower perceived informative value for the present research questions or their redundancy with the retained measures.

The second dimension, cognitive workload, addresses the extent to which disassembly execution is characterized by demanding cognitive processes [19]. To achieve this, mean pupil diameter, blink rate, and low/high index of pupillary activity (LHIPA) introduced by Duchowski et al. [37] were measured. The mean pupil diameter was included as a tonic indicator of workload-related changes in pupil size. The blink rate was selected as an exploratory workload-related measure, acknowledging that blink behavior may reflect overlapping processes, including fatigue, attentional suppression, and transient disengagement. LHIPA was utilized as a phasic, pupil-based index intended to capture short-term changes in pupillary activity. The relevance of these measures is predicated on the assumption that disassembly in conditions of uncertainty or low routinization is expected to increase processing demands.

The third dimension, scanpath structure, captures the spatial and sequential organization of gaze shifts across the visual scene without any information about the allocated gaze point to areas of interest [38]. It was operationalized using mean step length, revisit rate, and recurrence rate. The mean step length is defined as the average spatial distance between subsequent fixation locations, thus indicating whether visual exploration is concentrated in a localized manner or distributed across a wider range of regions. The revisit rate is defined as the extent to which gaze returns to locations that have previously been inspected. It can be interpreted as an indicator of repeated checking, uncertainty, or verification behavior. The recurrence rate is defined as the extent to which the scanpath is repetitive and provides information about the extent to which gaze follows cyclical or repeated inspection patterns. This dimension was considered especially relevant for the present study, since disassembly often requires repeated confirmation of product conditions, visual inspection of previous manipulation areas, and adaptation to uncertain conditions. Scanpath-based measures have been shown to provide direct information regarding whether visual behavior is organized in a focused and progressive manner or whether it becomes more repetitive and inspection-heavy [39].

The fourth dimension, gaze organization and entropy, addresses the degree of order, regularity, and predictability in gaze behavior. It was represented by spatial gaze entropy (SGE) and gaze transition entropy (GTE). SGE quantifies the spatial distribution of gaze across the visual field, reflecting whether gaze is concentrated in a limited number of regions or distributed more broadly across the scene. GTE has been demonstrated to be a reliable indicator of the unpredictability of transitions between gaze locations. This is indicative of the degree of sequential organization in visual exploration. Entropy-based measures were considered due to their capacity to provide a concise representation of the regularity versus irregularity of gaze behavior. This is particularly relevant for the differentiation of routine-like forms of task execution. Lower entropy values are interpreted as indicative of more structured and predictable gaze behavior, whereas higher entropy values are suggestive of a broader, less predictable, or less organized pattern of visual exploration.

The implementation of these four dimensions is intended to address two overarching purposes. Firstly, it reduces redundancy among individual eye-tracking metrics by grouping them into conceptually related categories. Secondly, it facilitates a theory-driven interpretation of disassembly-related gaze behavior by linking each metric to a specific aspect of manual action control. Moreover, the adoption of this dimensional approach enabled the formulation of expected directions of effects (see Table 1). Situations involving tasks that are more demanding or less routine-like were expected to be associated with more intensive visual sampling, more repeated checking, broader gaze shifts, and greater workload-related pupil responses. Conversely, it was hypothesized that higher levels of skill would be associated with more stable and more structured gaze behavior. Consequently, the selected metrics were not regarded as interchangeable outcomes, but rather as complementary indicators of different aspects of visually guided manual action.

## 3. Materials and Methods

### 3.1. Participants

A total of 46 healthy participants (22 females, 24 males) between the ages of 19 and 30 years (*M* = 24.31, *SD* = 2.68) were enrolled in this study. The sample consisted primarily of university students from the Department of Mechanical Engineering with no visual impairments or neurological conditions and who reported normal vision. Prior to participation, each participant was provided with comprehensive information regarding the study, and each participant signed a declaration of consent. The research study was approved by the Ethics Committee of Karlsruhe Institute of Technology.

### 3.2. Task and Procedure

**Figure 1:**
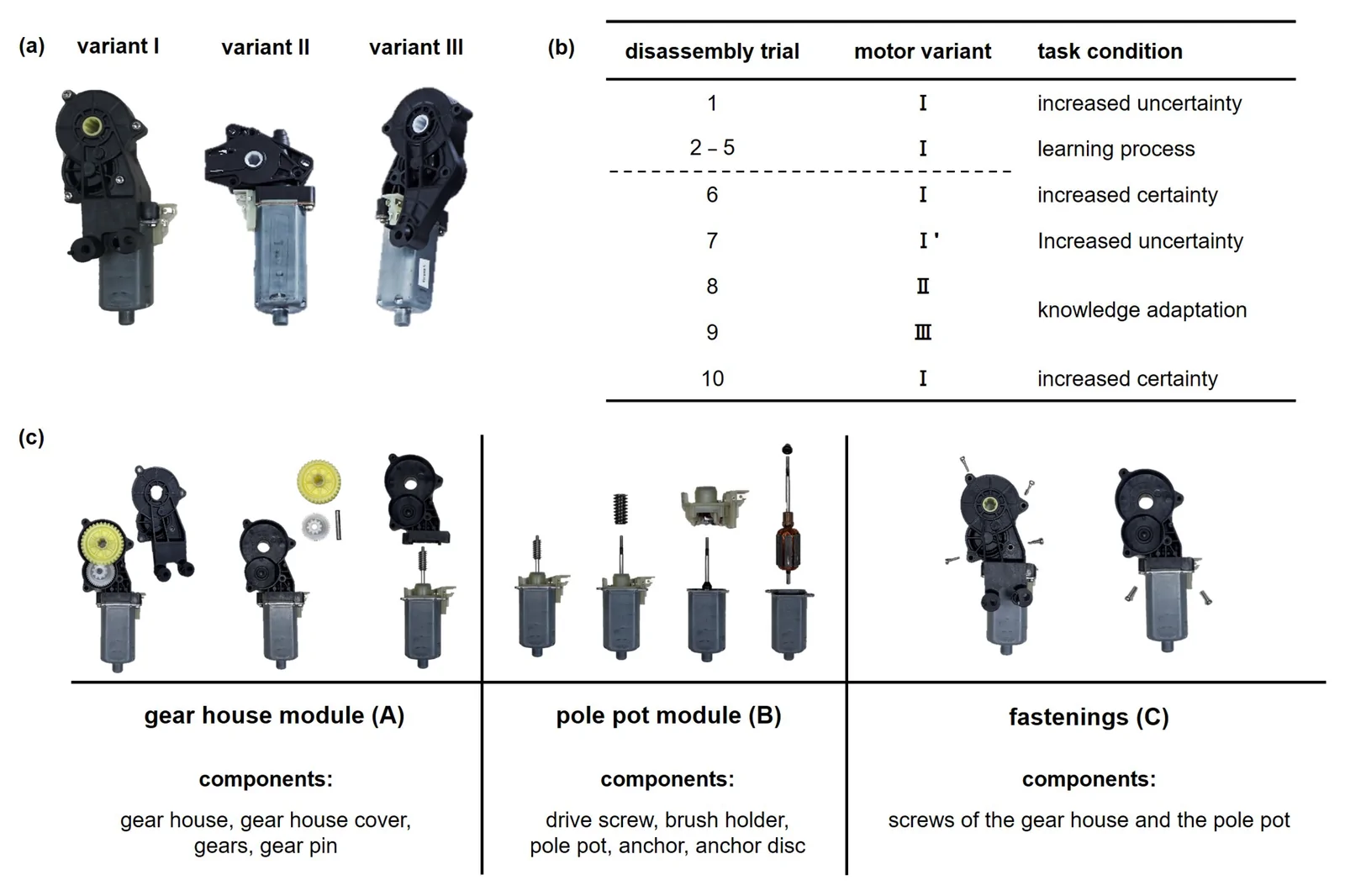
Overview of the experimental design, disassembly object and task conditions. (**a**) Variants of the electric servomotor used as the disassembly object. (**b**) Task conditions across repeated trials. The sequence demonstrates the manner in which the participants proceeded from an initial state of uncertainty to repeated execution and subsequent adaptation to modified task demands. (**c**) Modules and individual components of the electric motor removed during the disassembly procedure.

**Figure 2:**
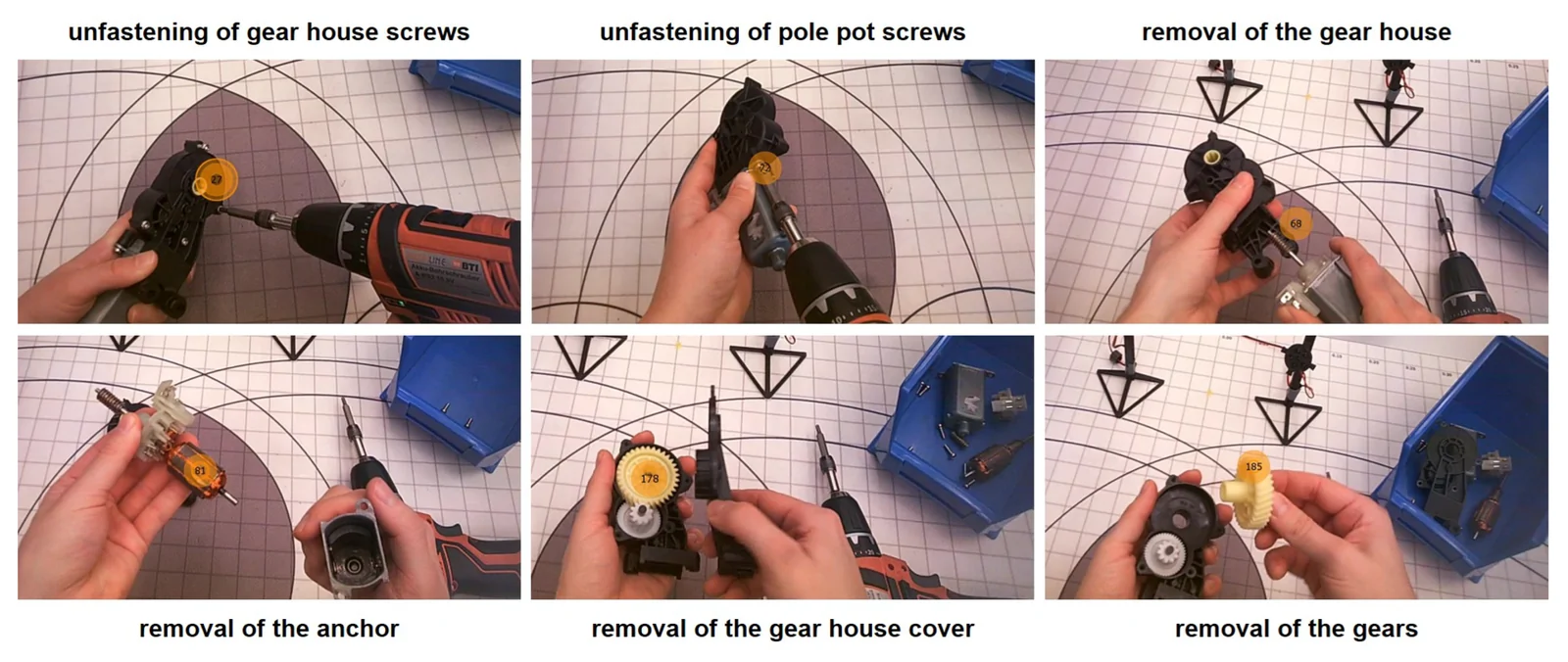
Illustration of manual disassembly processes, including the unfastening of screws and the removal of components, from the first-person perspective with the visualized gaze point.

Participants were tasked with the challenge of completely disassembling a small electric servomotor. Across the experiment, ten instances of motors were presented, encompassing three variants (see Figure 1a). Furthermore, each motor comprised ten individual components (see Figure 1c), which were organized into three distinct functional modules (gear house, pole pot, fastenings). For each trial, participants had to (i) unscrew the fastening screws belonging to module C, which secure the gear house cover to the gear house and the pole pot to the gear house, and (ii) remove the subsequent components: gear house, gear house cover, gears, gear pin, drive screw, brush holder, anchor, anchor disc and pole pot (see Figure 1c). The participants were instructed to disassemble the entire motor, using only an electric screwdriver. Figure 2 illustrates instances of different manual disassembly processes from the participant’s first-person point of view and the visualized gaze point. However, during the experiment, all motor components were found to be without faults, with the exception of one component that exhibited a standardized malfunction: a jammed gear inserted into one motor of variant I. This fault remained undetected until the gear cover was removed and the participant attempted to remove the gears, since it was not visible from the outside. This resulted in an unanticipated problem-solving scenario. After completing the first six disassembly procedures for variant I of the electric motor, variants I’ with the standardized defect, II and III were disassembled in a randomized order (see Figure 1b). The main difference between variants II and III was in the dimensions of the gear housing. The participants rapidly identified and resolved the disassembly steps to be undertaken. In instances where components had not been disassembled, the supervisor provided participants with general information that more components could be disassembled. This one-time reminder was initially offered after the second trial and repeated at the beginning of subsequent trials. If participants failed to successfully disassemble the electric motor, the entire procedure was explained after the fifth trial at the latest.

The establishment of the specific disassembly sequence was conducted in accordance with four different task conditions, analogous to processes in remanufacturing. These comprised increased uncertainty, learning process, increased certainty and knowledge adaptation. The first trial was conducted without prior knowledge of motor variant I or the necessary disassembly steps, resulting in increased uncertainty. In the subsequent four trials (2–5), only the same variant I of the motor as in trial 1 was disassembled. This enabled the participants to leverage their existing knowledge, thus categorizing these trials as learning processes. Following the completion of these trials, each participant was required to take a five-minute break without any specified tasks. Then, in trial six, the same motor variant I was disassembled to investigate the application of existing knowledge. This was defined as the first disassembly procedure under increased certainty. In the subsequent three trials, the motor variants I’, II and III were randomly disassembled. It was assumed that variant I’ with the standardized malfunction was disassembled with increased uncertainty, whereas the participants were required to find feasible problem-solving strategies. To facilitate a successful disassembly process for variants II and III, existing knowledge needed to be adapted. Consequently, this condition was classified as knowledge adaptation. In the final trial, participants were required to retrieve their established knowledge and disassemble variant I of the motor once more under increased certainty. Following the completion of the disassembly procedures, participants were requested to complete a questionnaire. In addition to the gathering of demographic data, participants rated their existing handcraft skills or the experience they had in handcraft. There were four possible items: (i) no prior experiences; (ii) medium prior experiences (familiarity with screwdrivers, pliers, etc.); (iii) extensive prior experiences (as a do-it-yourself enthusiast); and (iv) professional experience. According to the subjective self-assessment, the eye and gaze metrics can be analyzed in relation to differences between skill levels.

### 3.3. Recording Device

Eye and gaze data were recorded with the SMI Eye Tracking Glasses 2w, a mobile binocular system operating at 60 Hz. The device provides a gaze tracking accuracy of 0.5° and a field of view of 80° horizontal and 60° vertical. The front scene camera operates at a resolution of 1280 × 720 and a frame rate of 30 Hz. The calibration procedure was executed using the built-in one-point calibration supplied by the SMI ETG software and was repeated between each trial. The raw eye-tracking signals were processed using the analysis software SMI BeGaze (version 3.6.40). The software utilizes a dispersion-based detection algorithm for saccades, fixations, and blinks. A saccade is defined as a rapid change in gaze location, and a fixation is regarded as being bordered by two saccades. Blink detection can be considered a special case of fixation in which the presence of eye data is absent. The integrated low-speed event detection algorithm first searches for fixation events using a dispersion-based algorithm. Subsequently, saccade events are computed and derived from the primary fixation event. The algorithm identifies fixations as groups of consecutive points within a particular dispersion or maximum separation. In accordance with the findings of Salvucci et al. [40], the minimum duration was set to 80 ms, and the maximum dispersion to 100 pixels.

### 3.4. Data Preprocessing

Prior to conducting statistical analyses to answer the stated research questions, the exported eye and gaze data underwent a systematic preprocessing procedure [41]. The preprocessing and calculation of the selected eye and gaze metrics were conducted using MATLAB 2025b. To calculate the pupil diameter, the pupil signal was preprocessed [42,43] by removing blink- and artifact-contaminated samples, interpolating missing values using piecewise cubic spline interpolation, and smoothing the interpolated signal with a Savitzky–Golay filter (third-order polynomial, 7-sample window; 60 Hz sampling rate). The LHIPA was computed as a wavelet-based metric using a modified approach based on Duchowski et al. [37]’s implementation. The preprocessed pupil signal was linearly detrended, segmented into overlapping windows of one-second length, and decomposed using a discrete wavelet transform (sym16). Following this, it was transformed into a ratio of lower- to higher-frequency detail coefficients. Local maxima of this ratio were identified and thresholded using a universal thresholding rule, and the number of retained events per second was used as the LHIPA estimate. Prior to the analysis of the metrics of the scanpath structure, the recorded fixation coordinates were smoothed by a third-order moving median in order to reduce the influence of small head movements and measurement noise on gaze-position-based metrics. Subsequently, scanpath length and repeated fixation behavior were computed using the smoothed coordinates. The purpose of this procedure was to attenuate high-frequency positional jitter, which had the potential to artificially increase estimated gaze travel distance or the number of apparent returns to previously viewed locations. As no explicit head-motion compensation or transformation into a world-referenced coordinate system was applied, the smoothing should be considered a pragmatic noise-reduction step rather than a full correction of head-movement effects. Given that scanpath length is determined by the number of fixation transitions and trial duration, the normalized mean step length was derived for between-subject comparison. For repeated fixation behavior, revisit rate and recurrence rate were used as proportion-based measures that are less sensitive to differences in total fixation count. A revisit was defined as a fixation falling within 50 pixels of a previously visited location. Finally, SGE and GTE were computed using a fixed-grid discretization of the visual field. A single grid resolution of 4 × 4 was selected for the dataset using pooled gaze data and subsequently held constant across all participants and conditions to ensure comparability. SGE was calculated as the Shannon entropy [44] of the spatial occupancy distribution, whereas GTE was calculated as the conditional entropy of transitions between successive spatial states. Both measures were additionally normalized by the maximum possible entropy for the selected grid.

### 3.5. Statistical Analysis

Statistical analyses were conducted in R (v4.5.2) using a linear mixed-effect modeling approach to account for the repeated-measures structure of the data, with multiple trial-level observations nested within participants. Core packages utilized for the analysis comprised dplyr [45], tidyr [46], lme4 [47], and ggplot2 [48]. Within the dataset, task duration was retained as a continuous variable and additionally z-standardized prior to modeling to improve interpretability and numerical stability in interaction models. Standardized task duration was used as an indirect behavioral indicator of less routine-like execution. This interpretation is based on the assumption that longer disassembly trials indicate reduced action fluency and increased searching, hesitation or problem-solving. However, task duration is not a direct measure of routinization. It may also be influenced by factors such as individual working speed, motor skills, temporary distractions, and differences in manipulation complexity. Therefore, effects related to duration are cautiously interpreted as indicators of process fluency rather than as direct evidence of routine formation. Because the selected eye and gaze metrics differed in scale and distribution, normalization for statistical modeling was performed on a variable-specific basis. Positively skewed rate-like variables, including fixation rate, blink rate, and LHIPA, were log-transformed. Proportion-like variables, including revisit rate and recurrence rate, were logit-transformed after bounding values away from 0 and 1 by a small constant. Metrics with approximately continuous unbounded distributions, such as mean fixation duration, mean saccade amplitude, mean pupil diameter, mean step length, SGE, and GTE, were analyzed in their original scale. The decision to implement this normalization approach on a variable-specific basis was based on the finding that z-standardization, while effective in rescaling variables, is insufficient to address skewness, boundedness, and mean–variance dependency [49]. For each dimension (see Table 1), metric-specific linear mixed-effect models were fitted and interpreted jointly at the dimension level. Participant-specific random intercepts were included in all models, and additional random slopes were considered if supported by the data and model convergence. For categorical predictors with more than two levels, post hoc pairwise comparisons were performed using estimated marginal means with Holm correction for multiple comparisons. In order to control the false discovery rate across multiple model-based tests, the Benjamini–Hochberg FDR correction [50] was additionally applied to the resulting *p*-values.

## 4. Results

In total, the data analysis comprised 43 out of the 46 participants. This decision was necessitated by the incomplete recording of eye and gaze data, as well as low data quality, which resulted in the exclusion of the results from three participants from the dataset. The subjective self-assessment of prior handcraft skill experience yielded the following responses: no prior experience (2), medium prior experience (21), extensive prior experience (17), and professional experience (3). Due to the minimal number of self-ratings allocated to those with no experience or professional experience and to ensure a more balanced representation of prior experience, the group was assigned into two groups, the none/medium (23) and extensive/professional (20) skill level. For readability purposes, the none/medium handcraft experience group is referred to as novice and the extensive/professional handcraft experience group is referred to as expert in the statistical reporting. These terms refer only to lower and higher self-assessed handcraft experience, not to verified professional disassembly expertise. Furthermore, as demonstrated in Table 2, the largest numbers of incomplete disassembly trials were identified in the initial two trials. However, following the fourth trial, each participant was able to disassemble the entire motor. For the purposes of the present study, only data from complete disassembly trials were considered.

**Figure 3:**
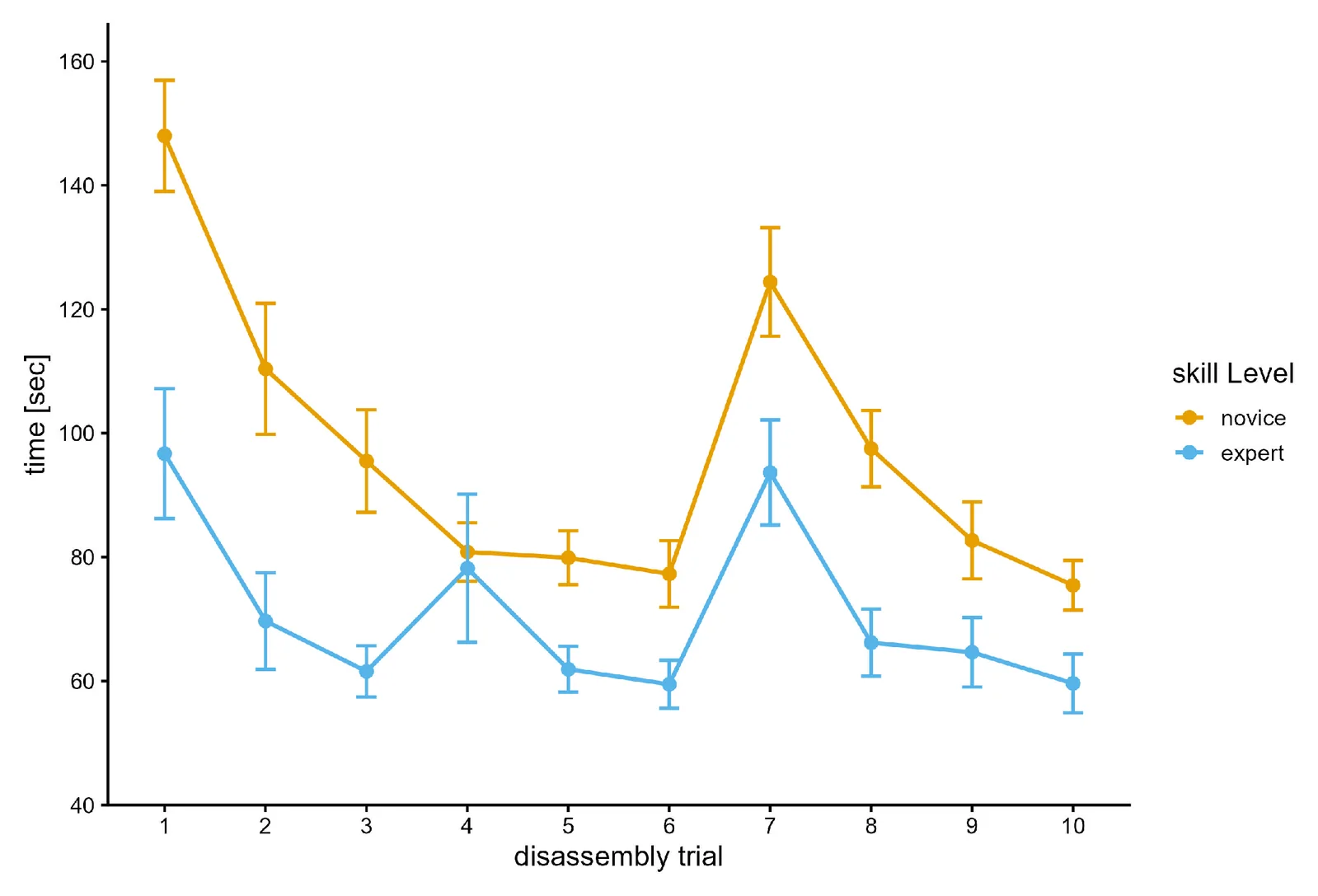
Mean disassembly duration is illustrated across the ten disassembly trials, distinguishing between novices and experts. Notably, the results are based on the duration from complete disassembly trials, with the points denoting the mean of each trial and the error bars indicating the standard error of the mean.

Furthermore, Figure 3 illustrates the mean disassembly duration across the ten disassembly trials, relative to the grouping of novices and experts by the self-assessed skill level. The results indicated that the two trials (1 and 7), which represented the task condition of increased uncertainty, required the longest duration. Therefore, it can be concluded that the time required for the disassembly procedures in the absence of prior knowledge or an unforeseen defect is comparable. Additionally, a gradual decrease in duration was observed in the task condition of the learning process (trials 2–5), with the exception of trial 4 from the experts. The two trials conducted under conditions of increased certainty (trial 6 and 10) resulted in the most time-efficient disassembly. Trials 8 and 9, which focused on knowledge adaptation and involved disassembling two different variants of the electric motor, did not reveal any particular findings regarding the duration of the disassembly process. As demonstrated by these findings, the inclusion of trial number as a control variable and a covariate in the following fitted linear mixed-effect models is considered to be important to address the defined research questions.

### 4.1. RQ1: How Do Different Task Conditions Affect Eye and Gaze Behavior During Human Disassembly?

**Figure 4:**
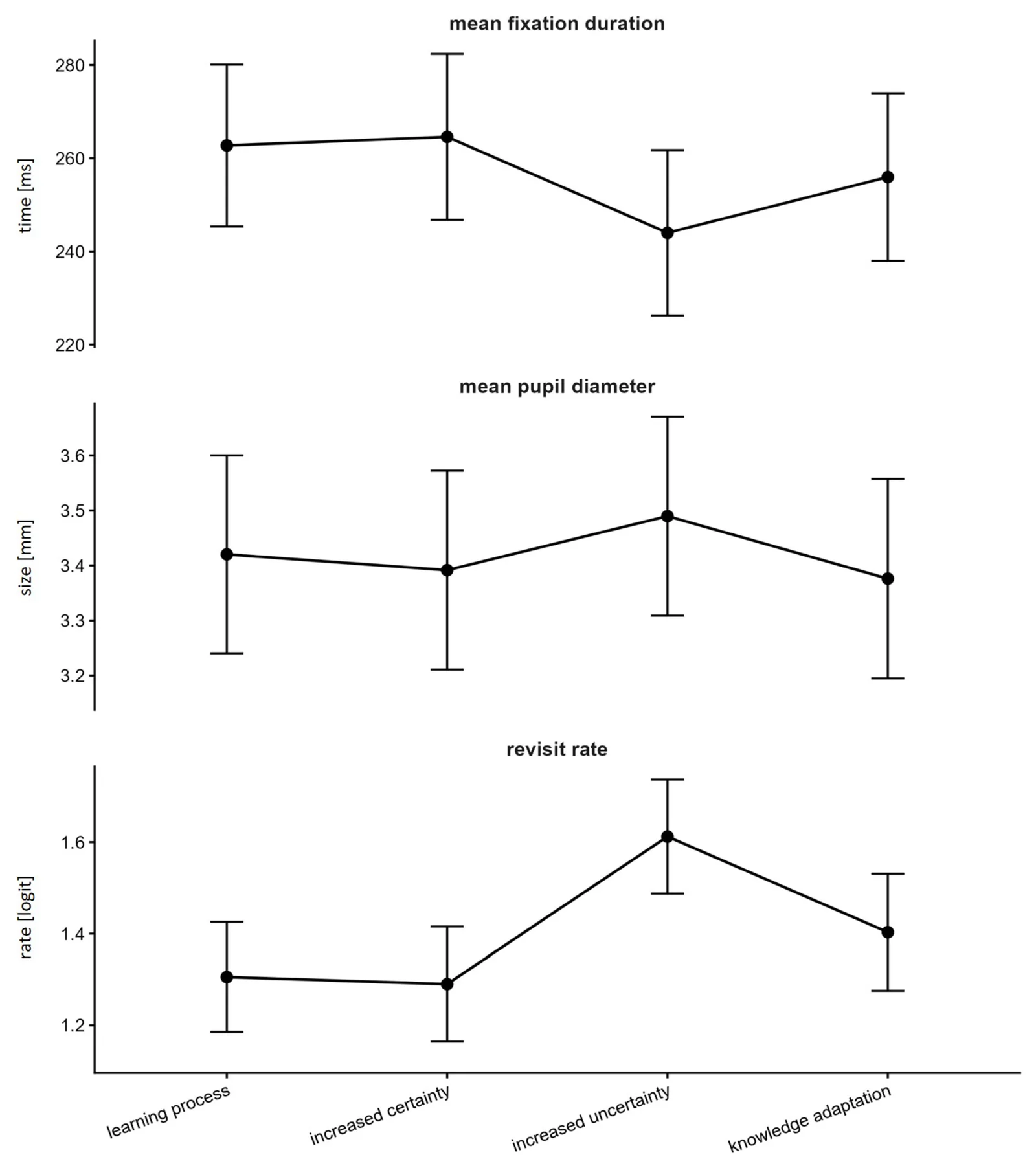
Estimated marginal means and 95% confidence intervals for the primary effects of significant eye and gaze metrics by task condition. Shown are model-adjusted values for mean fixation duration, mean pupil diameter, and revisit rate. The increased uncertainty condition was associated with shorter fixation durations, larger pupils, and higher revisit rates than the baseline learning-process condition.

To address this research question, linear mixed-effect models were fitted for each metric, with task condition as the primary predictor, trial number as a covariate, and participants as random intercepts. Significant effects of task condition were found for mean fixation duration, fixation rate, mean pupil diameter and revisit rate (see Figure 4). Following the implementation of a correction for multiple comparisons, no reliable task-condition effects were observed for the remaining metrics. For full transparency, the complete results of the corresponding models are reported in Table A1 and Table A4 in the Appendix A.

In the dimension of visual attention allocation, mean fixation duration differed significantly across task conditions, F(3, 355.19)=8.97, $p_{adj}<0.001$. In relation to the reference condition of the learning process, the increased uncertainty condition was associated with significantly shorter fixation durations (β=−18.74, SE=4.12, t=−4.54, $p_{adj}<0.001$, d=−0.31, 95% CI [−26.84,−10.63]). No significant differences from the reference condition were found for increased certainty or knowledge adaptation. Post hoc comparisons confirmed that fixation durations were shorter in increased uncertainty than in both learning process ($p_{adj}=0.001$) and increased certainty ($p_{adj}=0.008$). A comparable pattern was found for fixation rate, which also varied significantly across task conditions, F(3, 355.30)=7.34, $p_{adj}=0.001$. Compared with the condition of the learning process, increased uncertainty was associated with a significantly higher fixation rate (β=0.05, SE=0.01, t=4.21, $p_{adj}<0.001$, d=0.32, 95% CI [0.03, 0.07]). Again, no reliable differences were found for increased certainty, whereas knowledge adaptation showed only a trend-level increase. Mean saccade amplitude did not vary significantly across task conditions, F(3, 352.63)=0.01, p=0.999. Within the cognitive workload dimension, mean pupil diameter showed a significant effect of task condition, F(3, 355.03)=7.72, $p_{adj}<0.001$. Relative to the learning process condition, the increased uncertainty condition was associated with significantly larger pupils (β=0.07, SE=0.02, t=3.51, $p_{adj}=0.001$, d=0.12, 95% CI [0.03, 0.11]). Post hoc comparisons further showed that pupil diameter in increased uncertainty exceeded that in learning process ($p_{adj}=0.019$), increased certainty ($p_{adj}=0.008$), and knowledge adaptation ($p_{adj}=0.003$). Neither blink rate, F(3, 355.38)=2.20, p=0.088, nor LHIPA, F(3, 398.00)=0.20, p=0.895, showed significant task-condition effects. With regard to the scanpath structure, the strongest task-condition effect was found for revisit rate, F(3, 355.14)=27.17, $p_{adj}<0.001$. Relative to the learning process condition, the increased uncertainty condition showed a substantially higher revisit rate (β=0.31, SE=0.04, t=8.13, $p_{adj}<0.001$, d=0.68, 95% CI [0.23, 0.38]). Post hoc comparisons confirmed that revisit rate in increased uncertainty exceeded that in learning process ($p_{adj}<0.001$), increased certainty ($p_{adj}<0.001$), and knowledge adaptation ($p_{adj}=0.003$). In contrast, recurrence rate was not affected by task condition, F(3, 355.15)=0.65, p=0.583. Finally, no significant task-condition effects were observed for gaze organization and entropy measures. Task condition did not significantly affect SGE, F(3, 355.54)=0.70, p=0.552, or GTE, F(3, 355.48)=0.22, p=0.880. Thus, the task-condition manipulation primarily affected fixation dynamics, pupil size, and revisit behavior rather than global entropy-based gaze measures. At the model level, the variance explained by fixed effects alone was modest across RQ1 models (marginal $R^{2}$ values ranging from 0.002 to 0.077), whereas conditional $R^{2}$ values were markedly higher (0.555 to 0.947), indicating substantial between-subject variability in eye and gaze behavior.

### 4.2. RQ2: How Are Process Dynamics Reflected in Eye and Gaze Behavior During Disassembly as Indicated by Skill Level and Indicators of Routine-like Forms?

To examine mean-level skill differences (RQ2a), linear mixed-effect models were fitted for each metric with skill level as the predictor, trial number as a covariate, and participants as random intercepts. After correction for multiple testing, no significant main effects of skill level were found in any of the four conceptual dimensions (see Table A2 in the Appendix A). Thus, novices and experts did not differ reliably in their eye and gaze behavior across the selected metrics. This pattern was also reflected in the model fit indices (see Table A5 in the Appendix A). The variance explained by the fixed effects was small across models (marginal $R^{2}$ values ranging from 0.002 to 0.038), whereas conditional $R^{2}$ values were substantially higher (0.202 to 0.944), indicating that a considerable proportion of variance was attributable to between-participant differences rather than to skill level itself. Because RQ2a was also based on the assumption that higher skill would be associated with more stable and less variable gaze behavior, supplementary variability analyses were conducted. For this purpose, participant-level coefficients of variation across trials were calculated for each metric and compared between skill groups using linear models (see Table A7 in the Appendix A). At the nominal level, only the variability of mean fixation duration differed between groups, F(1, 41)=4.76, p=0.035, $\eta^{2}=0.104$. However, this effect did not remain significant after FDR correction ($p_{adj}=0.418$). No significant skill-level differences were found for the variability of fixation rate, saccade amplitude, pupil diameter, blink rate, LHIPA, revisit rate, recurrence rate, SGE, GTE, or task duration (${\mathrm{time}}_{z}$) after correction. Overall, these analyses did not provide robust evidence that experts showed more stable gaze behavior than novices.

**Figure 5:**
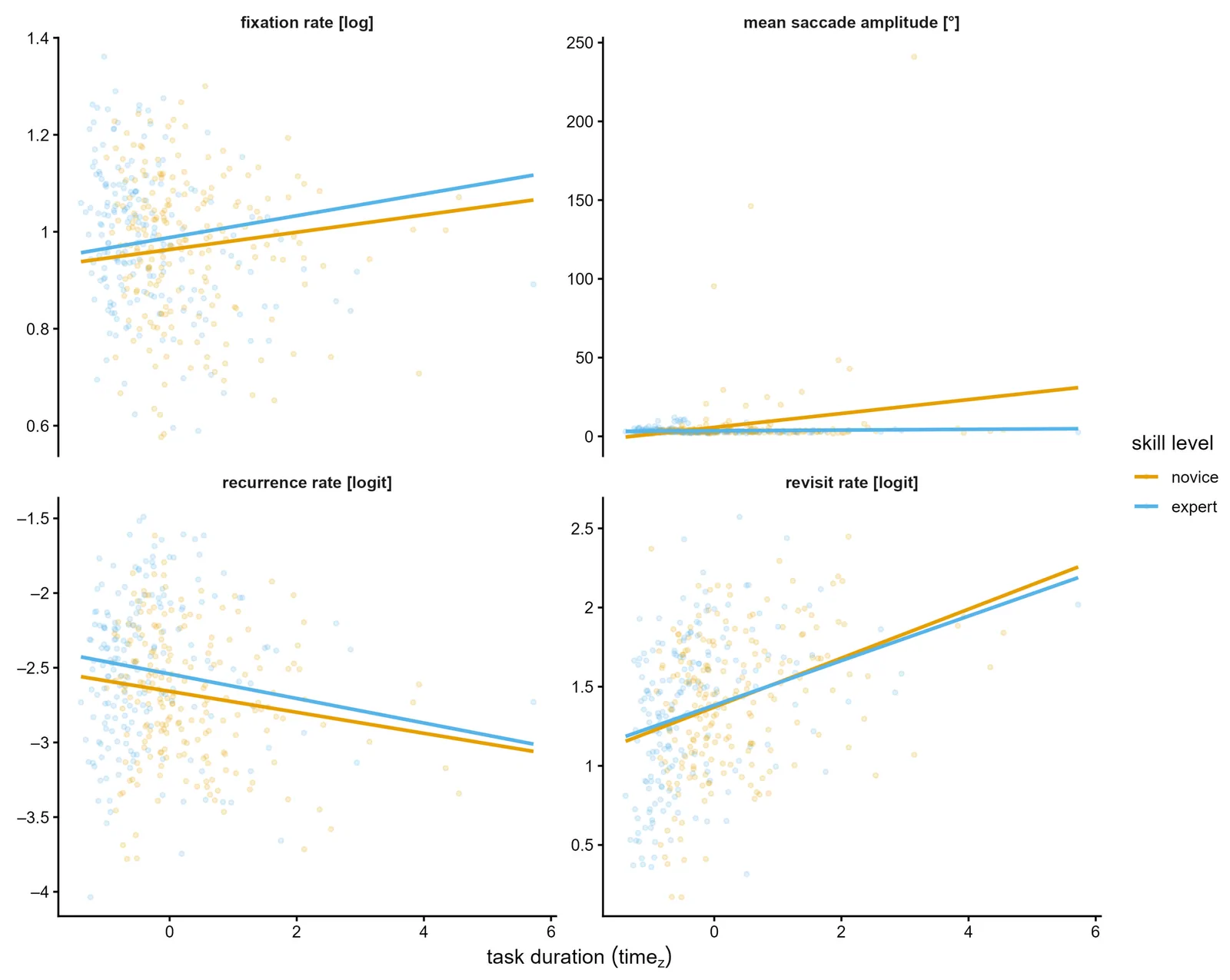
Predicted effects of standardized task duration (${\mathrm{time}}_{z}$) and model-based regression lines for fixation rate, mean saccade amplitude, revisit rate, and recurrence rate. Longer task duration, used as an indicator of less routine-like forms of task execution, was associated with more active visual sampling and altered scanpath structure.

To address variations within associated indicators for a routine-like form of disassembly (RQ2b), linear mixed-effect models were fitted with skill level, standardized task duration (${\mathrm{time}}_{z}$), their interaction, and trial number as predictors. Due to the unavailability of a direct quantitative classification of routinization, longer task duration was treated as an indicator of less routine-like task execution, while trial number captured repetition-related adaptation across repeated performance. Across the models, the most consistent effects were associated with task duration, whereas main effects of skill level and interactions with skill level were generally weak (see Figure 5). In the dimension of visual attention allocation, longer task duration was associated with shorter mean fixation duration, F(1, 370.52)=22.84, $p_{adj}<0.001$, higher fixation rate, F(1, 374.50)=14.04, $p_{adj}=0.002$, and larger mean saccade amplitude, F(1, 341.55)=7.42, $p_{adj}=0.045$. The corresponding fixed effects indicated that increasing task duration was associated with shorter fixations (β=−8.26, SE=2.52, t=−3.28, $p_{adj}=0.004$, d=−0.14, 95% CI [−13.21,−3.31]), more frequent sampling (β=0.02, SE=0.01, t=2.67, $p_{adj}=0.027$, d=0.12, 95% CI [0.01, 0.03]), and broader gaze shifts (β=4.41, SE=1.10, t=4.02, $p_{adj}<0.001$, d=0.29, 95% CI [2.25, 6.56]). The interaction between skill level and ${\mathrm{time}}_{z}$ was significant for mean saccade amplitude, F(1, 344.73)=6.16, $p_{adj}=0.043$, indicating that the positive relationship between duration and saccade amplitude was weaker in experts than in novices. Within the cognitive workload dimension, no reliable duration-related effects were found for mean pupil diameter, blink rate, or LHIPA. Thus, less routine-like execution was not robustly reflected in the workload-related measures. With regard to the scanpath structure, strong duration-related effects emerged. Longer task duration was associated with a significantly higher revisit rate, F(1, 385.44)=62.96, $p_{adj}<0.001$, with a fixed effect of β=0.16, SE=0.02, t=6.65, $p_{adj}<0.001$, d=0.34, 95% CI [0.11, 0.20]. At the same time, longer duration was associated with a significantly lower recurrence rate, F(1, 383.95)=14.30, $p_{adj}=0.020$, with β=−0.07, SE=0.03, t=−2.80, $p_{adj}=0.020$, d=−0.15, 95% CI [−0.12, −0.02]. These findings indicate that longer trials, used here as an indirect indicator of less routine-like execution, were characterized by more repeated checking but lower sequential stability of scanpaths. Finally, within the dimension of gaze organization and entropy, SGE and GTE showed only nominal effects of task duration, with F(1, 390.71)=5.19, p=0.023 and F(1, 388.80)=5.27, p=0.022, respectively. Following the application of the FDR correction, neither effect remained statistically significant ($p_{adj}=0.117$).

Overall, these results suggest that less routine-like forms of disassembly were primarily reflected in fixation dynamics and scanpath structure rather than in pupil-based workload measures or global entropy measures. Marginal $R^{2}$ values ranged from 0.012 to 0.114, with the largest fixed-effect contribution observed for revisit rate, whereas conditional $R^{2}$ values ranged from 0.218 to 0.944. For full transparency, the complete results of the corresponding models are reported in Table A3 and Table A6 in the Appendix A.

## 5. Discussion

The present study examined whether specific eye and gaze metrics reflect differences in disassembly conditions and process dynamics, considering influences on performance such as skill level and routine-like forms of task execution. Multidimensional analyses of visual attention, cognitive workload, scanpath structure, gaze organization, and entropy revealed that gaze behavior was significantly influenced by task-related uncertainty and by changes associated with task duration, whereas expertise had a limited effect. Specifically, under conditions of increased uncertainty fixation rate, mean pupil diameter and revisit rate increased, while longer tasks modulated fixation dynamics, saccade amplitude, revisit rate, and recurrence rate. Conversely, the self-assessed skill level of the participants did not yield consistent differences in mean gaze measures or variability after correcting for multiple comparisons. Furthermore, entropy-based indices showed no significant condition effects and exhibited only weak, non-robust relations to duration. Nevertheless, the findings of this study demonstrate that global eye and gaze metrics can be reliably used to reflect process-level variations in task execution, even in complex conditions. However, the same global metrics provide only limited discrimination between self-reported skill levels, suggesting that broad, process-oriented indices are more effective for detecting state-related changes in task control than for capturing trait-like differences among operators in this context.

### 5.1. Task Uncertainty as a Factor in Visual Sampling and Verification Behavior

A central result of the study was that increased task uncertainty was associated with shorter fixation durations, higher fixation rates, larger mean pupil diameters, and higher revisit rates. This pattern suggests that in uncertain disassembly situations, participants engaged in intensified visual sampling, increased processing demands, and more frequent re-inspection of previously viewed locations. In relation to research findings on fixation behavior, it can be interpreted that uncertainty primarily altered the temporal dynamics of visual sampling [51]. In situations where the conditions were perceived as uncertain, the participants demonstrated a tendency to engage in shorter and more frequent fixations. This behavior could be interpreted as an indication that the participants were attempting to gather more information about the environment. In the context of the present disassembly task, this pattern may indicate a necessity to review visual information more rapidly across multiple regions or to verify object states relevant to action repeatedly. This interpretation is consistent with the increase in revisit rate, which suggests more frequent returns to locations that have previously been inspected. The observed variation in pupil size is consistent with this result. Consequently, the present finding should be regarded as convergent rather than as stand-alone evidence. Increased uncertainty is more robustly interpreted due to the consistency observed across fixation-based, pupil-based, and scanpath-based measures. The results of the study demonstrate that uncertainty in disassembly is reflected to a lesser extent in a single gaze marker than in a more extensive pattern of gaze changes. This finding provides support for the utilization of a multidimensional approach in the analysis of eye and gaze movements in dynamic manual tasks. Furthermore, the practical significance of these effects should also be considered. While several task-condition effects demonstrated statistical significance, their practical significance should be interpreted with caution. In the context of the increased uncertainty condition, the impact on mean fixation duration was small to moderate, with a reduction relative to the learning-process condition (d=−0.31). A similarly small-to-moderate effect was observed for fixation rate, which was higher under increased uncertainty (d=0.32). The effect on mean pupil diameter was statistically reliable but small (d=0.12), suggesting that changes in workload related to uncertainty were detectable but limited in magnitude. In contrast, the most significant effect was observed for revisit rate, which increased substantially under conditions of elevated uncertainty (d=0.68). This signifies a practically relevant increase in repeated visual checking behavior. Therefore, the practical relevance of the findings lies in the consistent pattern across fixation dynamics, pupil response and scanpath behavior, rather than in large effects across all individual metrics. These results suggest that uncertainty in disassembly is most clearly reflected in repeated inspection behavior, while other gaze-based indicators show smaller, complementary changes.

As previously mentioned, the study focused solely on the calculation of global eye and gaze metrics. While this facilitates the drawing of conclusions regarding the overall disassembly process, it does not provide a temporal analysis of eye and gaze behavior during individual action phases. From an applied perspective, this information can provide insights into the actual cognitive state of the operator and can enable a more comprehensive interpretation of disassembly behavior. Given that the disassembly process frequently involves non-standardized product conditions and uncertain component states, operators must adapt their actions in a highly dynamic manner. Consequently, the present findings can be used as indicators to detect action phases with higher uncertainty, leading to conclusions about the component state or enhanced visual sampling and re-inspection, thus facilitating the planning of problem-solving strategies. Such information therefore has the potential to enable the development of adaptive assistance systems that provide context-sensitive support during disassembly, for example, by highlighting likely problem regions or adjusting information presentation when visual re-inspection behavior increases. Future research should consider a comprehensive time-series analysis of eye and gaze data [52] to obtain this information.

### 5.2. Limited Differences Between Skill Groups

Contrary to the initial assumption, no significant differences were observed between the lower and higher self-assessed handcraft experience groups after correction for multiple testing. This was observed in both the mean gaze measures and the analyses of variability across disassembly trials. Therefore, the current data does not provide substantial evidence that the selected global eye and gaze metrics reliably distinguish the two self-assessed skill groups under the tested conditions. This non-significant result may be explained, at least in part, by the limited operationalization of expertise. Disassembly expertise is likely to develop through repeated exposure to a variety of products, component conditions, tools, and failure modes in industrial remanufacturing environments. In contrast, the present participants were mainly young university students and were grouped according to self-assessed general handcraft experience. This approach may not capture the perceptual-motor routines that characterize professional disassembly operators. Furthermore, the impact of expertise on gaze behavior may manifest more potently in temporally and spatially specific measures, such as anticipatory fixations, transitions in gaze between tools and components, or fixation timing relative to manual actions, rather than in global trial-level averages. Consequently, the absence of significant skill-level effects should not be interpreted as evidence that expertise is irrelevant for disassembly. Rather, it is indicative of the limitations of the current skill proxy and global metrics in detecting such differences. To derive skill-relevant operator strategies, future empirical studies should concentrate more on a sample of workers from the remanufacturing industry, especially employees of disassembly stations. Utilizing such field observations, it is feasible to capture the eye and gaze behavior of operators with extensive experience and to analyze more stable patterns that represent such expertise.

### 5.3. Routine-like Execution Was Reflected Primarily in Search Dynamics and Scanpath Structure

Regarding process dynamics, longer task duration was used as an indirect indicator of less routine-like forms of task execution. Across the linear mixed-effect models, duration-related effects were found to be more consistent than effects related to skill level. Longer task duration, cautiously interpreted as an indirect proxy of less fluent and potentially less routine-like execution, was associated with shorter fixation durations, higher fixation rates, larger saccade amplitudes, higher revisit rates and lower recurrence rates. This pattern suggests that extended trials involved more intensive visual sampling and less stable scanpath organization. However, it does not provide a direct, process-level classification of routinization. Participants in longer trials demonstrated a higher frequency of visual information sampling, a wider range of gaze shifts, and a greater tendency to return to locations previously inspected. Simultaneously, the lower recurrence rate suggests that these extended trials were not merely more repetitive in a structured sense, but rather less stable in their sequential gaze organization. This distinction is crucial. Revisit rate and recurrence rate both relate to repeated gaze behavior, but they appear to capture different aspects of visual control. In the present study, it was demonstrated that an increased duration was associated with a greater frequency of repeated checking, but with a reduced stability of scanpath structure. This suggests that re-inspection without a robust or repetitive pattern of eye movement is associated with less routine-like execution. Conversely, the duration-related effects for pupil-based workload and entropy measures were found to be marginal and inconsistent following multiple-testing correction. Thus, in the present dataset, less routine-like forms of execution were reflected more clearly in fixation and scanpath measures than in workload- or entropy-based metrics. This aspect is of particular relevance for the purpose of learning from human disassembly behavior, with the objective of enhancing robotic and semi-automated systems. If there is a reliable association between less routine-like execution and more frequent revisits, broader gaze shifts, and more intensive visual sampling, these patterns may serve as observable markers of uncertainty and process instability. In future human–robot applications, such markers could facilitate the identification of deviations from routine behavior by human operators, thereby indicating the necessity for additional support. Furthermore, these markers could assist in identifying task segments that are likely to be difficult to automate directly without richer perceptual control.

## 6. Limitations and Future Work

Several limitations must be considered. Firstly, the differentiation between more routine-like and less routine-like execution was based on indirect indicators as compared to an independently labeled process classification. While task duration and skill level provide a plausible approximation, they do not constitute a direct quantitative ground truth. Secondly, the absence of robust skill-level effects can be partly attributed to the way in which skill was operationalized. Participants were categorized based on their self-assessed general handcraft experience, rather than on an objective measure of disassembly competence or professional experience in remanufacturing. This distinction is important because the effects of expertise on eye movement behavior are typically most pronounced when expertise is domain-specific and when tasks reflect the actual perceptual and motor demands of the expert domain [53]. The present grouping may therefore have been too broad to capture stable perceptual-motor strategies relevant to disassembly. Future research should encompass objective skill assessments, standardized pre-tests, or samples of industrial remanufacturing operators. Thirdly, the analysis was based on global gaze metrics, with no consideration given to action-linked or object-specific measures. As a result, the findings inform overall process dynamics, but not fine-grained gaze allocation to specific components or manipulation phases. Furthermore, it has been determined that specific measures, including LHIPA and entropy-based metrics, may be more dependent on preprocessing choices and discretization parameters than fixation- or scanpath-based measures. Consequently, their relative insensitivity in the present results should be interpreted with caution and may necessitate further methodological validation. A further limitation concerns the exclusion of incomplete disassembly trials. Most of these occurred in the early trials, particularly during the initial uncertainty and learning phases. Excluding these trials increased comparability across observations, as all analyzed trials contained the full disassembly sequence. However, this decision may have reduced the impact of factors such as uncertainty, task failure, and early problem solving. Future analyses could treat incomplete trials as a separate behavioral outcome, combine completion status with gaze metrics, or use phase-specific analyses that allow partial trials to be included up to the point at which the procedure was terminated. Finally, the generalizability of the findings is limited by the sample of participants and the laboratory setting. The sample consisted primarily of young university students, and the task involved disassembling a controlled electric motor rather than a real industrial remanufacturing process. Industrial disassembly often involves greater product variability, contamination, tool diversity, time pressure, ergonomic constraints and organizational routines. Therefore, the present findings should be interpreted as laboratory-based evidence of the feasibility of using global eye and gaze metrics to characterize disassembly behavior, rather than as direct evidence of industrial operator behavior. Future research should explore these results in more depth and in a specific direction. As discussed, to analyze eye and gaze metrics on a more fine-grained and temporal level, comprehensive time-series analysis is required. In addition, action-linked or object-specific measures, including the allocation of gaze points to areas of interests (AOIs), should be integrated into this process. This will facilitate a sophisticated analysis of eye and gaze behavior during human disassembly and the determination of action sequences and strategies related to task conditions. In order to concentrate on the impact of skill level on eye and gaze behavior, it is necessary to establish a reliable, robust and objective measure. This could encompass an analysis of experienced workers at a disassembly station, a standardized training procedure in a future study, or a detailed, standardized aptitude test of handcraft skills. A final methodological limitation is that the standardized malfunction was restricted to one defect type in one motor variant, specifically a jammed gear in variant I’. While this facilitated a controlled problem-solving scenario, subsequent studies should encompass a range of defect types, locations and randomly generated fault conditions across variants to ascertain whether the observed eye and gaze indicators of uncertainty generalize to a more extensive array of disassembly problems.

## 7. Conclusions

In summary, the present study demonstrates that specific eye and gaze metrics were sensitive to variations in task condition and process dynamics during manual disassembly. Increased uncertainty was primarily associated with intensified visual sampling, increased pupil diameter, and a greater frequency of inspection. Less routine-like forms of execution were reflected mainly in fixation dynamics and scanpath structure. In contrast, robust differences between self-assessed skill groups were not observed. Overall, the findings indicate that global gaze metrics are a viable method for capturing state-related variation in visually guided manual behavior, particularly in uncertain and non-routine task conditions. Moreover, from a broader perspective, this is of particular relevance to the field of remanufacturing and circular-economy systems, wherein efficient disassembly represents a crucial foundation for the subsequent reprocessing. The present findings do not directly evaluate adaptive assistance systems or robotic disassembly. However, they suggest that certain eye and gaze metrics could serve as potential indicators of uncertainty and reduced process fluency during manual disassembly. If these indicators are validated in industrial settings and combined with action or object-specific information, they could contribute to the development of context-sensitive assistance systems and human–robot interaction concepts in the future.

## Figures and Tables

**Table 1:** Selected eye and gaze metrics, their conceptual dimensions, and their assumed relevance to the research questions.

Dimension	Metric	Unit	RQ1	RQ2a	RQ2b
visual attention allocation	mean fixation duration	ms	P	S	S
	fixation rate	no./s	P	S	S
	mean saccade amplitude	°	S	S	S
cognitive workload	mean pupil diameter	mm	P	S	P
	blink rate	no./s	S	–	S
	LHIPA	–	S	S	P
scanpath structure	mean step length	px	P	P	P
	revisit rate	%	P	P	P
	recurrence rate	%	S	P	P
gaze organization and entropy	spatial gaze entropy (SGE)	bits	P	P	P
	gaze transition entropy (GTE)	bits	P	P	P

Note: P = primary relevance; S = supportive relevance; – = not primarily relevant. RQ1 addresses differences between disassembly conditions. RQ2a addresses skill-related differences in the stability and variability of gaze behavior. RQ2b addresses routine-like forms of disassembly behavior, operationalized indirectly via self-assessed skill and task duration.

**Table 2:** Quantitative results of complete and incomplete disassembly procedures across the first five trials, differentiated by novices and experts.

Trial	Variant	Complete		Incomplete	
		Novice	Expert	Novice	Expert
1	I	14	15	9	5
2	I	17	15	6	5
3	I	22	17	1	3
4	I	22	20	1	-
5	I	23	20	-	-

## Data Availability

The original contributions presented in this study are included in the article. Further inquiries can be directed to the corresponding author.

## Appendix A

This appendix contains supplementary results of the fixed-effect estimates and model fit comparisons for the fitted linear mixed-effect models, respectively, for RQ1, RQ2a, and RQ2b. In addition, the results of the one-way ANOVA used in the variability analyses within RQ2a are presented.

**Table A1:** Fixed-effect estimates of RQ1.

Metric	Parameter	β	SE	t	*p*	$p_{\mathrm{adj}}$	Cohen’s *d*	95% CI
mean fixation duration	(Intercept)	264.260	8.919	29.628	<0.001 ***	<0.001 ***		[246.725, 281.796]
	increased certainty	1.842	5.250	0.351	0.726	0.858	0.030	[−8.481, 12.164]
	increased uncertainty	−18.735	4.124	−4.543	<0.001 ***	<0.001 ***	−0.306	[−26.842, −10.628]
	knowledge adaptation	−6.766	5.546	−1.220	0.223	0.431	−0.110	[−17.669, 4.137]
	trial num	−0.262	0.836	−0.314	0.754	0.869	−0.012	[−1.905, 1.380]
fixation rate	(Intercept)	0.973	0.021	46.714	<0.001 ***	<0.001 ***		[0.932, 1.014]
	increased certainty	0.005	0.014	0.351	0.726	0.858	0.034	[−0.023, 0.032]
	increased uncertainty	0.046	0.011	4.211	<0.001 ***	<0.001 ***	0.320	[0.025, 0.068]
	knowledge adaptation	0.029	0.015	1.934	0.054	0.157	0.197	[0.000, 0.058]
	trial num	−0.002	0.002	−1.092	0.276	0.505	−0.047	[−0.007, 0.002]
mean saccade amplitude	(Intercept)	2.237	2.099	1.065	0.287	0.514		[1.891, 6.364]
	increased certainty	0.184	2.525	0.073	0.942	0.963	0.012	[−4.780, 5.148]
	increased uncertainty	−0.140	1.982	−0.070	0.944	0.963	−0.009	[−4.036, 3.757]
	knowledge adaptation	0.168	2.666	0.063	0.950	0.964	0.011	[−5.075, 5.410]
	trial num	0.520	0.400	1.300	0.194	0.403	0.095	[−0.267, 1.308]
mean pupil diameter	(Intercept)	3.418	0.089	38.368	<0.001 ***	<0.001 ***		[3.243, 3.593]
	increased certainty	−0.029	0.025	−1.139	0.255	0.487	−0.048	[−0.078, 0.021]
	increased uncertainty	0.069	0.020	3.509	<0.001 ***	0.002 **	0.116	[0.031, 0.108]
	knowledge adaptation	−0.044	0.027	−1.658	0.098	0.238	−0.074	[−0.096, 0.008]
	trial num	0.000	0.004	0.094	0.925	0.964	0.002	[−0.008, 0.008]
blink rate	(Intercept)	−1.610	0.148	−10.845	<0.001 ***	<0.001 ***		[−1.902, −1.318]
	increased certainty	−0.244	0.124	−1.968	0.050 *	0.151	−0.236	[−0.487, 0.000]
	increased uncertainty	0.076	0.097	0.785	0.433	0.674	0.074	[−0.115, 0.268]
	knowledge adaptation	−0.162	0.131	−1.236	0.217	0.425	−0.157	[−0.419, 0.095]
	trial num	0.036	0.020	1.832	0.068	0.183	0.097	[−0.003, 0.075]
LHIPA	(Intercept)	1.667	0.025	66.080	<0.001 ***	<0.001 ***		[1.617, 1.716]
	increased certainty	0.015	0.035	0.416	0.677	0.834	0.077	[−0.054, 0.084]
	increased uncertainty	0.018	0.028	0.645	0.519	0.728	0.094	[−0.036, 0.072]
	knowledge adaptation	0.002	0.037	0.064	0.949	0.964	0.012	[−0.071, 0.075]
	trial num	−0.002	0.006	−0.409	0.682	0.834	−0.033	[−0.013, 0.009]
mean step length	(Intercept)	37.693	1.256	30.013	<0.001 ***	<0.001 ***		[35.224, 40.162]
	increased certainty	1.250	1.007	1.242	0.215	0.425	0.144	[−0.729, 3.229]
	increased uncertainty	−0.649	0.791	−0.821	0.412	0.654	−0.075	[−2.204, 0.905]
	knowledge adaptation	0.809	1.063	0.761	0.447	0.680	0.093	[−1.281, 2.900]
	trial num	−0.286	0.160	−1.786	0.075	0.198	−0.091	[−0.601, 0.029]
revisit rate	(Intercept)	1.384	0.064	21.713	<0.001 ***	<0.001 ***		[1.259, 1.509]
	increased certainty	−0.015	0.048	−0.322	0.748	0.869	−0.034	[−0.110, 0.079]
	increased uncertainty	0.307	0.038	8.134	<0.001 ***	<0.001 ***	0.678	[0.233, 0.381]
	knowledge adaptation	0.098	0.051	1.924	0.055	0.157	0.216	[−0.002, 0.197]
	trial num	−0.014	0.008	−1.773	0.077	0.200	−0.083	[−0.029, 0.002]
recurrence rate	(Intercept)	−2.596	0.069	−37.547	<0.001 ***	<0.001 ***		[−2.731, −2.460]
	increased certainty	0.024	0.054	0.436	0.663	0.830	0.050	[−0.083, 0.130]
	increased uncertainty	−0.045	0.042	−1.054	0.292	0.516	−0.094	[−0.129, 0.038]
	knowledge adaptation	0.015	0.057	0.264	0.792	0.871	0.032	[−0.097, 0.127]
	trial num	−0.001	0.009	−0.018	0.942	0.964	−0.004	[−0.017, 0.016]
SGE	(Intercept)	0.404	0.014	28.019	<0.001 ***	<0.001 ***		[0.375, 0.432]
	increased certainty	−0.006	0.012	−0.452	0.652	0.831	−0.055	[−0.030, 0.019]
	increased uncertainty	−0.002	0.010	−0.249	0.803	0.877	−0.024	[−0.021, 0.016]
	knowledge adaptation	−0.017	0.013	−1.313	0.190	0.400	−0.170	[−0.043, 0.008]
	trial num	0.000	0.002	−0.029	0.977	0.977	−0.002	[−0.004, 0.004]
GTE	(Intercept)	0.290	0.011	27.449	<0.001 ***	<0.001 ***		[0.270, 0.311]
	increased certainty	−0.002	0.009	−0.195	0.846	0.910	−0.023	[−0.019, 0.015]
	increased uncertainty	0.001	0.007	0.158	0.875	0.933	0.015	[−0.012, 0.014]
	knowledge adaptation	−0.006	0.009	−0.648	0.517	0.728	−0.081	[−0.024, 0.012]
	trial num	−0.001	0.001	−0.468	0.468	0.697	−0.038	[−0.004, 0.002]

Note: * *p* < 0.05, ** *p* < 0.01, *** *p* < 0.001. N = 398 observations nested within 43 participants. The models were fitted for each metric with task condition as the predictor, trial number as a covariate, and participant as a random intercept: metric ∼ task condition + trial num + (1|participant). The reference category was the task condition learning process.

**Table A2:** Fixed-effect estimates of RQ2a.

Metric	Parameter	β	SE	t	*p*	$p_{\mathrm{adj}}$	Cohen’s *d*	95% CI
mean fixation duration	(Intercept)	251.809	11.756	21.419	<0.001 ***	<0.001 ***		[228.696, 274.922]
	skill level (expert)	15.045	16.209	0.928	0.354	0.582	0.246	[−16.822, 46.912]
	trial num	−0.145	0.540	−0.268	0.789	0.871	−0.007	[−1.206, 0.917]
fixation rate	(Intercept)	0.976	0.027	35.781	<0.001 ***	<0.001 ***		[0.922, 1.029]
	skill level (expert)	0.012	0.037	0.321	0.748	0.870	0.083	[−0.061, 0.085]
	trial num	−0.001	0.001	−0.857	0.391	0.637	−0.023	[−0.004, 0.001]
mean saccade amplitude	(Intercept)	3.861	2.210	1.747	0.081	0.208		[−0.484, 8.206]
	skill level (expert)	−3.611	2.392	−1.509	0.132	0.300	−0.238	[−8.314, 1.093]
	trial num	0.552	0.249	2.217	0.027 *	0.086	0.101	[0.062, 1.042]
mean pupil diameter	(Intercept)	3.397	0.122	27.734	<0.001 ***	<0.001 ***		[3.156, 3.638]
	skill level (expert)	0.121	0.174	0.694	0.488	0.712	0.202	[−0.221, 0.463]
	trial num	−0.007	0.003	−0.031	0.009 **	0.031 *	−0.031	[−0.012, −0.002]
blink rate	(Intercept)	−1.318	0.181	−7.290	<0.001 ***	<0.001 ***		[−1.674, −0.963]
	skill level (expert)	−0.397	0.237	−1.674	0.095	0.234	−0.385	[−0.864, 0.070]
	trial num	0.006	0.012	0.520	0.604	0.790	0.017	[−0.018, 0.031]
LHIPA	(Intercept)	1.653	0.024	69.674	<0.001 ***	<0.001 ***		[1.606, 1.699]
	skill level (expert)	0.037	0.019	1.956	0.051	0.152	0.195	[−0.000, 0.074]
	trial num	−0.002	0.003	−0.514	0.608	0.834	−0.026	[−0.009, 0.005]
mean step length	(Intercept)	38.702	1.545	25.042	<0.001 ***	<0.001 ***		[35.664, 41.741]
	skill level (expert)	−3.281	2.048	−1.602	0.110	0.262	−0.378	[−7.306, 0.745]
	trial num	−0.127	0.100	−1.272	0.204	0.417	−0.041	[−0.325, 0.070]
revisit rate	(Intercept)	1.508	0.082	18.348	<0.001 ***	<0.001 ***		[1.347, 1.670]
	skill level (expert)	−0.082	0.109	−0.752	0.452	0.681	−0.182	[−0.297, 0.132]
	trial num	−0.016	0.005	−2.950	0.003 **	0.013 **	−0.095	[−0.026, −0.005]
recurrence rate	(Intercept)	−2.698	0.086	−31.432	<0.001 ***	<0.001 ***		[−2.867, −2.529]
	skill level (expert)	0.165	0.114	1.441	0.150	0.332	0.348	[−0.060, 0.390]
	trial num	0.003	0.005	0.606	0.545	0.746	0.019	[−0.007, 0.014]
SGE	(Intercept)	0.416	0.018	23.403	<0.001 ***	<0.001 ***		[0.381, 0.451]
	skill level (expert)	−0.018	0.023	−0.776	0.438	0.694	−0.180	[−0.064, 0.027]
	trial num	−0.002	0.001	−1.344	0.180	0.383	−0.045	[−0.004, 0.001]
GTE	(Intercept)	0.297	0.013	22.468	<0.001 ***	<0.001 ***		[0.271, 0.323]
	skill level (expert)	−0.010	0.018	−0.559	0.576	0.770	−0.133	[−0.045, 0.025]
	trial num	−0.002	0.001	−1.851	0.065	0.182	−0.060	[−0.003, 0.000]

Note: * *p* < 0.05, ** *p* < 0.01, *** *p* < 0.001. N = 398 observations nested within 43 participants. The models were fitted for each metric with skill level as the predictor, trial number as a covariate, and participant as a random intercept: metric ∼ skill level + trial num + (1|participant). The reference category was the skill level of novices.

**Table A3:** Fixed-effect estimates of RQ2b.

Metric	Parameter	β	SE	t	*p*	$p_{\mathrm{adj}}$	Cohen’s *d*	95% CI
mean fixation duration	(Intercept)	256.879	12.144	21.153	<0.001 ***	<0.001 ***		[233.003, 280.754]
	skill level (expert)	8.835	16.778	0.527	0.599	0.790	0.144	[−24.153, 41.822]
	${\mathrm{time}}_{z}$	−8.259	2.518	−3.280	0.001 **	0.004 **	−0.135	[−13.210, −3.308]
	trial num	−0.582	0.530	−1.099	0.273	0.505	−0.026	[−1.623, 0.460]
	skill level:${\mathrm{time}}_{z}$	−2.844	3.992	−0.712	0.477	0.703	−0.046	[−10.694, 5.005]
fixation rate	(Intercept)	0.965	0.029	33.650	<0.001 ***	<0.001 ***		[0.909, 1.021]
	skill level (expert)	0.025	0.039	0.634	0.527	0.731	0.172	[−0.052, 0.102]
	${\mathrm{time}}_{z}$	0.018	0.007	2.665	0.008 **	0.027 *	0.124	[0.005, 0.031]
	trial num	0.000	0.001	−0.210	0.834	0.903	−0.006	[−0.004, 0.002]
	skill level:${\mathrm{time}}_{z}$	0.005	0.011	0.436	0.663	0.830	0.032	[−0.016, 0.025]
mean saccade amplitude	(Intercept)	1.719	2.191	0.785	0.433	0.674		[−2.588, 6.026]
	skill level (expert)	−2.188	2.319	−0.944	0.346	0.592	−0.144	[−6.748, 2.371]
	${\mathrm{time}}_{z}$	4.406	1.095	4.023	<0.001 ***	<0.001 ***	0.291	[2.253, 6.559]
	trial num	0.688	0.248	2.769	0.006 **	0.021 *	0.126	[0.199, 1.176]
	skill level:${\mathrm{time}}_{z}$	−4.175	1.682	−2.483	0.014 *	0.043 *	−0.276	[−7.481, −0.869]
mean pupil diameter	(Intercept)	3.403	0.123	27.747	<0.001 ***	<0.001 ***		[3.162, 3.644]
	skill level (expert)	0.114	0.174	0.653	0.514	0.728	0.191	[−0.229, 0.456]
	${\mathrm{time}}_{z}$	−0.008	0.012	−0.661	0.509	0.727	−0.014	[−0.033, 0.016]
	trial num	−0.007	0.003	−2.765	0.006 **	0.021 *	−0.034	[−0.012, −0.002]
	skill level:${\mathrm{time}}_{z}$	−0.006	0.020	−0.281	0.779	0.880	−0.009	[−0.045, 0.034]
blink rate	(Intercept)	−1.364	0.182	−7.506	<0.001 ***	<0.001 ***		[−1.721, −1.007]
	skill level (expert)	−0.341	0.237	−1.440	0.151	0.332	−0.331	[−0.807, 0.125]
	${\mathrm{time}}_{z}$	0.074	0.059	1.258	0.209	0.421	0.072	[−0.042, 0.189]
	trial num	0.010	0.013	0.829	0.407	0.655	0.028	[−0.014, 0.035]
	skill level:${\mathrm{time}}_{z}$	0.026	0.093	0.285	0.776	0.870	0.026	[−0.156, 0.208]
LHIPA	(Intercept)	1.647	0.025	67.060	<0.001 ***	<0.001 ***		[1.598, 1.695]
	skill level (expert)	0.039	0.020	1.976	0.049 *	0.152	0.207	[0.000, 0.078]
	${\mathrm{time}}_{z}$	0.015	0.014	1.097	0.273	0.505	0.078	[−0.012, 0.042]
	trial num	−0.001	0.003	−0.430	0.667	0.851	−0.022	[−0.008, 0.005]
	skill level:${\mathrm{time}}_{z}$	−0.022	0.020	−1.080	0.279	0.515	−0.114	[−0.060, 0.018]
mean step length	(Intercept)	38.471	1.539	24.991	<0.001 ***	<0.001 ***		[35.445, 41.498]
	skill level (expert)	−2.866	2.025	−1.415	0.158	0.342	−0.330	[−6.848, 1.115]
	${\mathrm{time}}_{z}$	0.276	0.477	0.579	0.563	0.760	0.032	[−0.662, 1.215]
	trial num	−0.102	0.102	−1.007	0.315	0.545	−0.033	[−0.302, 0.097]
	skill level:${\mathrm{time}}_{z}$	0.716	0.753	0.951	0.342	0.576	0.083	[−0.764, 2.197]
revisit rate	(Intercept)	1.420	0.079	18.003	<0.001 ***	<0.001 ***		[1.265, 1.575]
	skill level (expert)	0.012	0.105	0.111	0.912	0.964	0.026	[−0.194, 0.218]
	${\mathrm{time}}_{z}$	0.155	0.023	6.651	<0.001 ***	<0.001 ***	0.342	[0.109, 0.200]
	trial num	−0.008	0.005	−1.721	0.086	0.216	−0.052	[−0.018, 0.001]
	skill level:${\mathrm{time}}_{z}$	−0.014	0.037	−0.376	0.707	0.850	−0.031	[−0.086, 0.058]
recurrence rate	(Intercept)	−2.656	0.088	−30.324	<0.001 ***	<0.001 ***		[−2.828, −2.484]
	skill level (expert)	0.116	0.117	0.993	0.321	0.547	0.245	[−0.114, 0.346]
	${\mathrm{time}}_{z}$	−0.070	0.025	−2.796	0.006 **	0.020 *	−0.149	[−0.120, −0.021]
	trial num	0.000	0.005	−0.054	0.958	0.964	−0.002	[−0.054, 0.010]
	skill level:${\mathrm{time}}_{z}$	−0.012	0.040	−0.295	0.768	0.870	−0.025	[−0.090, 0.066]
SGE	(Intercept)	0.413	0.018	23.196	<0.001 ***	<0.001 ***		[0.378, 0.448]
	skill level (expert)	−0.011	0.023	−0.483	0.629	0.811	−0.112	[−0.057, 0.034]
	${\mathrm{time}}_{z}$	0.003	0.006	0.601	0.548	0.747	0.035	[−0.008, 0.015]
	trial num	−0.001	0.001	−1.003	0.316	0.546	−0.034	[−0.004, 0.001]
	skill level:${\mathrm{time}}_{z}$	0.014	0.009	1.546	0.122	0.287	0.140	[−0.004, 0.032]
GTE	(Intercept)	0.295	0.013	22.673	<0.001 ***	<0.001 ***		[0.269, 0.321]
	skill level (expert)	−0.005	0.017	−0.284	0.777	0.870	−0.066	[−0.038, 0.029]
	${\mathrm{time}}_{z}$	0.002	0.004	0.389	0.697	0.844	0.022	[−0.006, 0.010]
	trial num	−0.001	0.001	−1.512	0.131	0.299	−0.050	[−0.003, 0.000]
	skill level:${\mathrm{time}}_{z}$	0.012	0.006	1.834	0.067	0.182	0.161	[−0.001, 0.024]

Note: * *p* < 0.05, ** *p* < 0.01, *** *p* < 0.001. N = 398 observations nested within 43 participants. The models were fitted for each metric with skill level, standardized task duration (time_*z*_), their interaction, and trial number as predictors, as well as participant as a random intercept: metric ∼ skill level * time_*z*_ + trial num + (1|participant). The reference category was the skill level of novices.

**Table A4:** Model fit comparison of the linear mixed-effect models addressing RQ1.

Dimension	Metric	AIC	BIC	Marginal $R^{2}$	Conditional $R^{2}$
visual attention allocation	mean fixation duration	3953.26	3981.16	0.015	0.781
	fixation rate	−780.20	−752.30	0.016	0.720
	mean saccade amplitude	3270.86	3298.77	0.010	0.202
cognitive workload	mean pupil diameter	−228.67	−200.76	0.004	0.947
	blink rate	931.53	959.44	0.007	0.576
	LHIPA	−180.87	−152.97	0.002	NA
scanpath structure	mean step length	2604.48	2632.38	0.005	0.605
	revisit rate	189.85	217.76	0.077	0.673
	recurrence rate	278.74	306.65	0.002	0.622
gaze organization/entropy	SGE	−909.00	−881.09	0.004	0.555
	GTE	−1181.61	−1153.71	0.004	0.586

Note: The models are fitted through this formula for each dimension, for each eye and gaze metric: metric ∼ task condition + trial num + (1|participant). The reference category was the task condition learning process. Marginal $R^{2}$ denotes the proportion of variance explained by the fixed effects only, whereas conditional $R^{2}$ denotes the proportion of variance explained by both fixed and random effects. Lower AIC and BIC values indicate better relative model fit among comparable models. NA indicates that conditional $R^{2}$ could not be reliably estimated, likely because the random-effect variance was estimated at or near zero, resulting in a singular or boundary model fit.

**Table A5:** Model fit comparison of the linear mixed-effect models addressing RQ2a.

Dimension	Metric	AIC	BIC	Marginal $R^{2}$	Conditional $R^{2}$
visual attention allocation	mean fixation duration	3974.34	3994.27	0.016	0.764
	fixation rate	−762.92	−742.99	0.002	0.704
	mean saccade amplitude	3264.66	3284.59	0.024	0.202
cognitive workload	mean pupil diameter	−210.71	−190.78	0.011	0.944
	blink rate	931.36	951.29	0.038	0.568
	LHIPA	−188.07	−168.14	0.010	NA
scanpath structure	mean step length	2601.46	2621.39	0.038	0.601
	revisit rate	258.69	278.62	0.018	0.599
	recurrence rate	274.66	294.59	0.031	0.620
gaze organization/entropy	SGE	−911.50	−891.56	0.010	0.553
	GTE	−1185.25	−1165.32	0.008	0.586

Note: The models are fitted through this formula for each dimension, for each eye and gaze metric: metric ∼ skill level + trial num + (1|participant). The reference category was the skill level of novices. Marginal $R^{2}$ denotes the proportion of variance explained by the fixed effects only, whereas conditional $R^{2}$ denotes the proportion of variance explained by both fixed and random effects. Lower AIC and BIC values indicate better relative model fit among comparable models. NA indicates that conditional $R^{2}$ could not be reliably estimated, likely because the random-effect variance was estimated at or near zero, resulting in a singular or boundary model fit.

**Table A6:** Model fit comparison of the linear mixed-effect models addressing RQ2b.

Dimension	Metric	AIC	BIC	Marginal $R^{2}$	Conditional $R^{2}$
visual attention allocation	mean fixation duration	3956.35	3984.25	0.036	0.792
	fixation rate	−772.44	−744.53	0.019	0.740
	mean saccade amplitude	3252.92	3280.82	0.066	0.218
cognitive workload	mean pupil diameter	−207.91	−180.00	0.012	0.944
	blink rate	931.92	959.83	0.044	0.568
	LHIPA	−185.49	−157.58	0.014	NA
scanpath structure	mean step length	2602.34	2630.25	0.045	0.596
	revisit rate	200.61	228.51	0.114	0.654
	recurrence rate	264.46	292.37	0.052	0.645
gaze organization/entropy	SGE	−913.84	−885.94	0.024	0.556
	GTE	−1188.33	−1160.43	0.023	0.578

Note: The models are fitted through this formula for each dimension, for each eye and gaze metric: metric ∼ skill level * ${\mathrm{time}}_{z}$ + trial num + (1|participant). The reference category was the skill level of novices. Marginal $R^{2}$ denotes the proportion of variance explained by the fixed effects only, whereas conditional $R^{2}$ denotes the proportion of variance explained by both fixed and random effects. Lower AIC and BIC values indicate better relative model fit among comparable models. NA indicates that conditional $R^{2}$ could not be reliably estimated, likely because the random-effect variance was estimated at or near zero, resulting in a singular or boundary model fit.

**Table A7:** One-way ANOVA results comparing the selected eye and gaze metrics between skill levels.

Metric	Effect	df	Sum Sq	Mean Sq	F	*p*	$p_{\mathrm{adj}}$	Partial $\eta^{2}$
mean fixation duration	skill level	1, 41	600.811	600.811	4.763	0.035 *	0.418	0.104
	residuals	41	5171.607	126.137				
fixation rate	skill level	1, 41	0.001	0.001	1.402	0.243	0.584	0.033
	residuals	41	0.028	0.001				
mean saccade amplitude	skill level	1, 41	385.160	385.160	2.241	0.142	0.426	0.052
	residuals	41	7046.083	171.856				
mean pupil diameter	skill level	1, 41	0.004	0.004	0.721	0.401	0.601	0.017
	residuals	41	0.245	0.006				
blink rate	skill level	1, 41	0.203	0.203	0.744	0.393	0.601	0.018
	residuals	41	11.205	0.273				
LHIPA	skill level	1, 41	0.001	0.001	0.021	0.885	0.885	0.001
	residuals	41	0.254	0.006				
mean step length	skill level	1, 41	2.431	2.431	0.792	0.379	0.601	0.019
	residuals	41	125.882	3.070				
revisit rate	skill level	1, 41	0.003	0.003	0.501	0.483	0.644	0.012
	residuals	41	0.240	0.006				
recurrence rate	skill level	1, 41	0.001	0.001	0.180	0.673	0.808	0.004
	residuals	41	0.293	0.007				
SGE	skill level	1, 41	0.001	0.001	0.083	0.775	0.845	0.002
	residuals	41	0.021	0.001				
GTE	skill level	1, 41	0.001	0.001	2.546	0.118	0.426	0.058
	residuals	41	0.007	0.002				
${\mathrm{time}}_{z}$	skill level	1, 41	0.368	0.368	2.816	0.101	0.426	0.064
	residuals	41	5.362	0.131				

Note: * *p* < 0.05. df = degrees of freedom; Sum Sq = Sum of Squares; Mean Sq = Mean Square. The p-values are adjusted for multiple comparisons using the FDR correction. Partial *η*^2^ is reported as the measure of effect size. Residuals (error term) are reported to illustrate the model’s variance components. Skill-level groupings of experts and novices are based on participants’ self-assessments.
